# Hazardous use of benzodiazepine receptor agonists in psychiatric clinics in China: electronic prescription database study

**DOI:** 10.1192/bjo.2022.589

**Published:** 2022-10-18

**Authors:** Xiaomin Xu, Jiajun Xu, Chuanwei Li, Gang Wang, Wenzhe Wang, Yujian Ye, Yong Chen, Tieqiao Liu, Min Zhao, Xuyi Wang, Na Zhong, Haifeng Jiang

**Affiliations:** Shanghai Mental Health Center, Shanghai Jiao Tong University School of Medicine, Shanghai, China; Mental Health Centre, West China Hospital, Sichuan University, Chengdu, China; Suzhou Psychiatric Hospital, The Affiliated Guangji Hospital of Soochow University, Suzhou, China; Wuhan Mental Health Center, Wuhan, China; Department of Patient & Health Impact, Pfizer Inc., Collegeville, Pennsylvania, USA; Mental Health Institute, The Second Xiangya Hospital of Central South University, Changsha, China; Shanghai Mental Health Center, Shanghai Jiao Tong University School of Medicine, Shanghai, China; Shanghai Key Laboratory of Psychotic Disorders, Shanghai, China; and CAS Center for Excellence in Brain Science and Intelligence Technology, Chinese Academy of Sciences, Shanghai, China; Shanghai Mental Health Center, Shanghai Jiao Tong University School of Medicine, Shanghai, China; and Shanghai Key Laboratory of Psychotic Disorders, Shanghai, China

**Keywords:** Benzodiazepine receptor agonists, hazardous use, over-indication use, prescription database analysis, psychiatric population

## Abstract

**Background:**

Benzodiazepine receptor agonists (BZRAs) are commonly used clinically and data on their hazardous use from large populations of psychiatric patients is limited.

**Aims:**

To assess the current status of hazardous BZRA use and related factors in Chinese out-patient psychiatric settings.

**Method:**

The study included out-patients with at least one BZRA prescription from five psychiatric settings in east, central and west China in 2018. Demographic and prescription information were extracted from the electronic prescription database. We defined the co-occurrence of overdose and long-term use as hazardous use, and patients whose recorded diagnoses did not meet any indications approved by the Chinese Food and Drug Administration as over-indication users. Additionally, 200 hazardous users were randomly selected for follow-up interview to confirm the actual situation.

**Results:**

Among 720 054 out-patients, 164 450 (22.8%) had at least one BZRA prescription; 55.9% of patients were prescribed over-indication and 3% were defined as hazardous users. Multilevel multivariate regression analysis with hospital as a random effect showed that factors associated with hazardous use were older age (18–64 years: β = 0.018; 95% CI 0.013–0.023; >65 years: β = 0.015; 95% CI 0.010–0.021), male (β = 0.005, 95% CI 0.003–0.007), over-indication (β = 0.013, 95% CI 0.012–0.015), more out-patient visits (β = 0.006, 95% CI 0.006–0.006) and more visits to different doctors (β = 0.007, 95% CI 0.007–0.008); 98.5% of hazardous users (197/200) could not be contacted.

**Conclusions:**

BZRAs are commonly used and there is a relatively large proportion of over-indication users among Chinese psychiatric out-patients. However, only a small proportion of hazardous users were detected. The study highlights how to use prescription data to support improvements in clinical practice.

Benzodiazepine receptor agonists (BZRAs) are the most prescribed psychoactive drugs globally; they suppress neuronal activity by binding to GABA_A_ receptors and increasing chloride ion influx to hyperpolarise the neuron's membrane potentials.^1^ Therefore, they are used for treatment of insomnia, anxiety, panic disorder and epilepsy in the short term (usually 2–4 weeks) in psychiatric settings.^1–4^ However, BZRAs may cause many physical and mental adverse effects, including respiratory depression, cognitive impairment, falls and injuries (especially in elderly people), psychiatric symptoms and increased risk of suicide.^5–7^ Another main problem of chronic BZRA use is the rapid development of tolerance, withdrawal or dependence within weeks to months.^8^

Overdose and long-term use are inconsistent with clinical recommendations. Previous studies have shown that hazardous use of BZRAs is quite common, and it results in a premorbid status with high risk of negative consequences, which warrants an early intervention.^9^ Additionally, nearly half of long-term benzodiazepine (BZD) users use overdose hypnotics.^10^ The use of hypnotics has increased rapidly in recent years, an increase almost entirely attributable to a rise in medium- and long-term use regardless of indication.^11^

Hypnotics are one of the most used classes of drug in psychiatric practice, with 27.9–75.9% of patients with psychiatric disorders (e.g. schizophrenia, affective disorders) using them at some point.^12,13^ However, few large-population studies are available to enable understanding of hazardous use of BZRAs in psychiatric settings around the world or in China.^12^ BZRAs are tightly controlled in China and users tend to continue to refill their prescriptions regularly in hospitals, so prescription databases enable highly reliable individual-level analysis of drug consumption.^14^

This study aimed to identify real-world use of hypnotics in psychiatric settings and its related factors using hospital electronic prescription databases in China.

## Method

### Study design

This study was a multicentre, retrospective, cross-sectional study of the current status of BZRA use in Chinese psychiatric settings. The authors assert that all procedures contributing to this work comply with the ethical standards of the relevant national and institutional committees on human experimentation and with the Helsinki Declaration of 1975, as revised in 2008. All procedures involving human patients were approved by Shanghai Mental Health Center (permission number: 2019–22). Informed consent was not required because of the anonymity and minimal risk of this observational study.

### Participants and definition of BZRA

Inclusion criteria were psychiatric out-patients who were prescribed at least one BZRA in 2018. The definition of BZRA was in accordance with the World Health Organization's Anatomical Therapeutic Chemical (ATC) classification codes. Alprazolam, estazolam, lorazepam, oxazepam, midazolam, diazepam, clonazepam, nitrazepam were categorised as BZDs, whereas zolpidem, zopiclone, eszopiclone and zaleplon were categorised as Z-drugs.

### Data source

We used the electronic prescription databases provided by a convenience sample of five hospitals with the largest number of clinic visits in their own regions: two in the east, two in central and one in the west of China. Three of these hospitals (Shanghai Mental Health Center, Affiliated Guangji Hospital of Soochow University and Wuhan Mental Health Center) were the only tertiary mental hospitals in their regions. The other two hospitals (the Second Xiangya Hospital of Central South University and the West China Hospital of Sichuan University) were general hospitals with psychology departments that had the same medical level and regional influence as the mental hospitals. All five hospitals are tertiary hospitals in cities. Owing to the high-quality psychiatric services provided by these hospitals, local civilians tend to seek psychiatric help in these settings, which makes the sampled hospitals well representative of local real situations.

Data on electronic prescription dispensing of BZRAs in 2018 were extracted for these out-patient psychiatric settings. The databases contained information including: (a) de-identified unique patient ID, (b) demographic characteristics (e.g. gender, age), (c) diagnosis and prescription information on drug type, dose, duration and usage, and (d) unique physician ID.

### Index and measurements

#### Defined daily dose

The defined daily dose (DDD) is an internationally accepted standard for measuring drug consumption endorsed by the World Health Organization. We used it to compare BZRA consumption between regions.^15^

#### Arrays of individual-level cumulative daily dosage in diazepam milligram equivalents

**Fig. 1:**
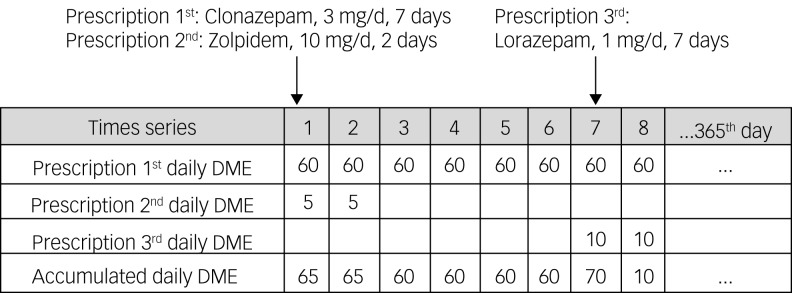
An example of a cumulative array of the daily dosage of hypnotics for one participant, converted to diazepam milligram equivalents (DMEs): 0.5 mg clonazepam equals 10 DMEs, 20 mg zolpidem equals 10 DMEs, 1 mg lorazepam equals 10 DMEs.

To compare the potency of different hypnotics, the dosages of different kinds of hypnotic were converted into diazepam milligram equivalents (DMEs).^15^ Then an individual-level array of cumulative DMEs was calculated day by day over the research period for each patient, which allowed comparison between patients (Fig. 1).

Based on these individual arrays of cumulative daily DME usage, the average daily dosage, total days of use and total yearly usage were calculated for each patient to give the annual hypnotics load. The number of out-patient visits and number of different doctors visited were calculated for further analysis.

#### Maximum averaged daily DME usage and overdose usage

Based on the arrays of cumulative daily DME usage, we found that some patients requested more hypnotics than justified by their conditions because they want to reduce the number of visits for repeat prescriptions. This might result in an overestimation of daily usage in our study. To avoid this, the 3-month period with most hypnotics use was identified and the average daily dosage in this period was calculated as the maximum daily DME dose. Also based on the arrays, patients were considered as overdose users if their maximum averaged daily DME dose was more than 40.

#### Maximum days of continuous use and long-term users (>90 days)

Based on the arrays of cumulative daily DME usage, the maximum number of days of continuous use of hypnotics was identified for each patient, and with permissible gap of up to 3 days because the withdrawn symptoms were most obvious in 72 h after the cessation. Withdrawal symptoms develop rapidly after 6 weeks of continuous use and tolerance shows within weeks to months;^16^ therefore guidelines recommend treatment periods of 2–4 weeks, depending on the indication.^1–4^ We chose a 90-day cut-off for long-term users^17^ to ensure at least three hypnotic prescriptions in a year, given the prescription length restrictions in China of no more than 30 days.

#### Hazardous hypnotics use

Based on the arrays of cumulative daily usage, patients with both long-term use of hypnotics (up to 90 days) and overdose use of hypnotics (over 40 mg) were considered to be patients with hazardous use of hypnotics. The other users were considered to be patients without hazardous use.

#### Over-indication use of hypnotics

Patients were considered to eb over-indication users if none of recorded diagnoses in 2018 met any of the indications approved by the Chinese Food and Drug Administration (FDA) for the specific drugs they were prescribed (see supplementary Table 1 available at https://dx.doi.org/10.1192/bjo.2022.589).

### Patient follow-up

We randomly selected 200 patients from the hazardous users in one of the hospitals in the east region, using simple randomisation tables generated by SPSS Statistics, and contacted them by phone. Trained psychiatrists conducted face-to-face semi-structured interviews with this selected sample about their use of BZRAs to ascertain whether they could be diagnosed with sedative hypnotics dependence according to ICD-11 criteria. Patients who received the follow-up interviews gave written consent.

### Statistical analysis

Statistical analysis was done in R on MacOS (version 3.6.2). The statistical significance level was *P* < 0.05. For the sociodemographic characteristics and the pattern of hypnotics use among participants, we used mean (s.d.) and median (interquartile range, IQR) for the continuous variables, and proportions for the categorical variables. For a very small proportion of participants, information on gender, age or diagnosis was invalid or missing, and we excluded these participants in further analyses.

Descriptive statistics were used to present data on the consumption of BZRAs described in DDD values, and the frequency and proportions of hazardous BZRA users in the five hospitals. The Kruskal–Wallis test was used to compare the difference in hypnotics consumption among different hospitals. The χ²-test or Wilcoxon rank test were used for the comparison of characteristics between patients with and without hazardous hypnotic use.

To examine the association between the variables of demographics and prescription characteristics and the risk of hazardous hypnotics use, we used multilevel multivariate regression analysis with hospital as a random effect as patients were nested within hospitals. Based on previous studies,^18–20^ we included the following patient variables that may be associated with hazardous BZRA use: age, gender, region, over-indication use, annual out-patient visits and the number of different doctors visited. Beta coefficients (β) and 95% confidence intervals (CIs) were presented for each potential predictor.

## Results

### Overall study population

Among the sample of 720 054 out-patients from five hospitals in China in 2018, 164 450 (22.8%) had at least one BZRA prescription (Table 1). There were significant between-hospital differences on all variables (*P* < 0.05). Most of the patients were female (63.6%) and were aged 18–64 (76.2%). The three most common diagnoses for hypnotics prescription were codes F30–F39 (32.7%), F40–F49 (33.5%) and G47 (28.2%).

**Table 1:** Characteristics of patients prescribed benzodiazepine receptor agonists in five hospitals in China

Variables	Total sample (*n* = 164 450)	East region		West region	Central region	
		Hospital A (*n* = 49 121)	Hospital B (*n* = 17 851)	Hospital C (*n* = 72 161)	Hospital D (*n* = 13 756)	Hospital E (*n* = 11 561)
Gender, *n* (%)						
Male	59 713 (36.3%)	17 537 (35.8%)	7119 (39.9%)	24 482 (33.9%)	6225 (45.3%)	4350 (37.6%)
Female	104 645 (63.6%)	31 507 (64.2%)	10 732 (60.1%)	47 678 (66.1%)	7520 (54.7%)	7208 (62.4%)
Age, years: *n* (%)						
<18	3651 (2.2%)	946 (2.0%)	270 (1.5%)	1721 (2.4%)	271 (2.0%)	443 (3.8%)
18–64	125 393 (76.2%)	35 655 (73.5%)	13 411 (75.1%)	55 421 (76.8%)	11 193 (81.7%)	9713 (84.0%)
≥65	31 671 (19.3%)	11 890 (24.5%)	4167 (23.3%)	15 017 (20.8%)	2235 (16.3%)	1405 (12.2%)
ICD-10 diagnosis codes, *n* (%)						
F01–F09	4048 (2.5%)	2351 (4.8%)	667 (3.7%)	759 (1.1%)	208 (1.5%)	63 (0.5%)
F10–F19	822 (0.5%)	176 (0.4%)	246 (1.4%)	215 (0.3%)	80 (0.6%)	75 (0.6%)
F20–F29	15 170 (9.2%)	6868 (14.0%)	2628 (14.7%)	2240 (3.1%)	2638 (19.2%)	796 (6.9%)
F30–F39	53 839 (32.7%)	18 981 (38.6%)	7445 (41.7%)	19 455 (27.0%)	4390 (31.9%)	3568 (30.9%)
F40–F49	55 134 (33.5%)	15 143 (30.8%)	5093 (28.5%)	28 432 (39.4%)	1796 (13.1%)	4670 (40.4%)
F50–F59	5041 (3.1%)	3783 (7.7%)	814 (4.6%)	63 (0.1%)	233 (1.7%)	148 (1.3%)
F60–F69	212 (0.1%)	125 (0.3%)	11 (0.0%)	5 (0.0%)	16 (0.1%)	55 (0.5%)
F70–F79	294 (0.2%)	189 (0.4%)	68 (0.4%)	26 (0.0%)	5 (0.0%)	6 (0.1%)
F80–F89	36 (0.0%)	20 (0.0%)	5 (0.0%)	0 (0.0%)	2 (0.0%)	9 (0.1%)
F90–F98	545 (0.3%)	149 (0.3%)	153 (0.9%)	120 (0.2%)	23 (0.2%)	100 (8.6%)
F99–F99	8854 (5.4%)	734 (1.5%)	6 (0.0%)	1093 (1.5%)	6661 (48.4%)	360 (3.1%)
G40–G41	1635 (1.0%)	67 (0.1%)	341 (1.9%)	1113 (1.5%)	94 (0.7%)	20 (0.2%)
G47	46 320 (28.2%)	274 (0.6%)	2341 (13.1%)	37 469 (51.9%)	3860 (28.1%)	2376 (20.6%)
Hazardous use, *n* (%)	4988 (3.0%)	2118 (4.3%)	491 (2.8%)	1995 (2.8%)	258 (1.9%)	126 (1.1%)
Maximum average daily DME usage, median (IQR)	4.4 (1.8–13.3)	5.0 (2.1–13.3)	4.3 (2.0–10.2)	5.9 (2.7–16.0)	0.6 (0.1–1.3)	1.7 (0.7–4.5)
Total DME dosage, median (IQR)	480 (160–2130)	567 (192–2267)	540 (180–1706)	700 (240–2640)	75 (13–1600)	150 (64–450)
Maximum continuous days of use, median (IQR)	33 (23–54)	33 (23–61)	39 (23–57)	38 (28–58)	7 (4–30)	17 (5–27)
Total days of use, median (IQR)	40 (21–110)	48 (24–120)	50 (24–126)	50 (30–123)	9 (2–45)	14 (4–28)
Number of out-patient visits, median (IQR)	3 (1–6)	4 (2–9)	4 (1–7)	3 (1–6)	2 (1–4)	1 (1–2)
Number of different doctors visits, median (IQR)	2 (1–4)	2 (1–5)	2 (1–4)	2 (1–4)	1 (1–4)	1 (1–1)
Over-indication use, *n* (%)	91 935 (55.9%)	35 591 (72.5%)	11 705 (65.6%)	28 819 (39.9%)	9975 (72.5%)	5845 (50.1%)

F01–F09: Organic, including symptomatic, mental disorders; F10–F19: Mental and behavioral disorders due to psychoactive substance use; F20–F29: Schizophrenia, schizotypal and delusional disorders; F30–F39: Mood [affective] disorders; F40–F49: Neurotic, stress-related and somatoform disorders; F50–F59: Behavioral syndromes associated with physiological disturbances and physical factors; F60–F69: Disorders of adult personality and behavior; F70–F79: Mental retardation; F80-F89: Disorders of psychological development; F90–F98: Behavioral and emotional disorders with onset usually occurring in childhood and adolescence; F99–F99: Unspecified mental disorder; G40–G41: Epilepsy and recurrent seizures; G47: Sleep disorders; DME,diazepam milligram equivalent.

### Patterns of BZRA prescription in the five hospitals

**Fig. 2:**
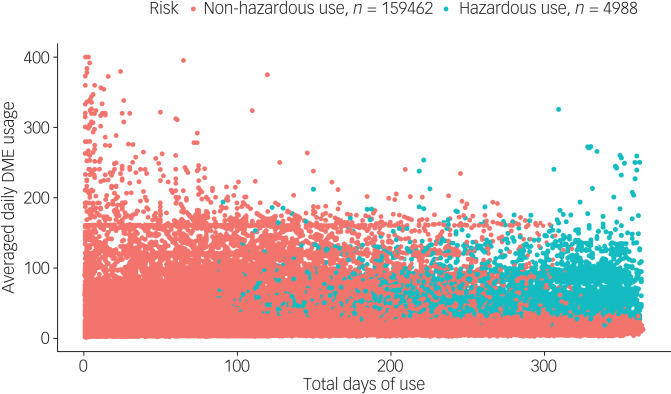
Scatter plot of the pattern of hypnotics use for all patients with non-hazardous and hazardous use. Each data point represents a patient and shows their total days of use and the corresponding average daily diazepam milligram equivalent (DME) usage.

**Fig. 3:**
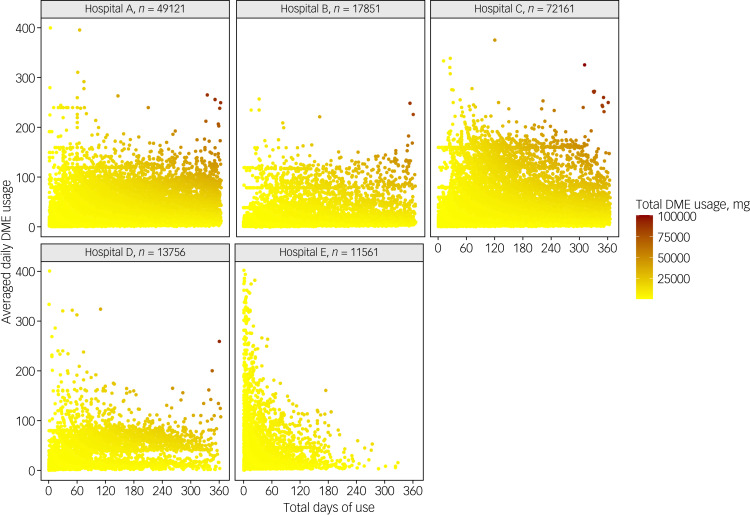
Scatter plot for pattern of hypnotics use in the five hospitals. Each data point represents a patient and shows their total days of use and the corresponding average daily diazepam milligram equivalent (DME) usage.

Most patients were medicated with relatively low average daily DME doses for short-term use, and only 4988 (3.0%) were defined as hazardous users (Fig. 2). The overall pattern of hypnotics use for each patient by hospital is depicted in Fig. 3. The patterns of BZRA use were similar in hospitals A, B, C and D, with 4.3%, 2.8% 2.8% and 1.9% of patients treated with hazardous prescriptions respectively. Although only 1.1% of patients in hospital E were hazardous users, there were still some patients prescribed extremely high average daily DME doses for relatively short periods (Fig. 3).

The median maximum average daily DME usage was 4.4 (IQR 1.8–13.3); the median total DME usage was 480 (IQR 160–2130); the median maximum continuous days of use was 33 (IQR 23–54); the median total days of use was 40 (IQR 21–110); the median number of out-patient visits was 3 (IQR 1–6); and the median number of different doctors visited was 2 (IQR 1–4). Patients in hospital C (the west region) showed the highest dosage and longest duration of use, whereas those in hospitals D and E (the central region) showed the lowest. Moreover, patients in hospitals A and B (the east region) had a larger number of out-patient visits and visits to different doctors compared with patients in other hospitals. Over-indication use occurred in 55.9% of the total patient sample, with the largest proportion (72.5%) in hospitals A and D.

**Fig. 4:**
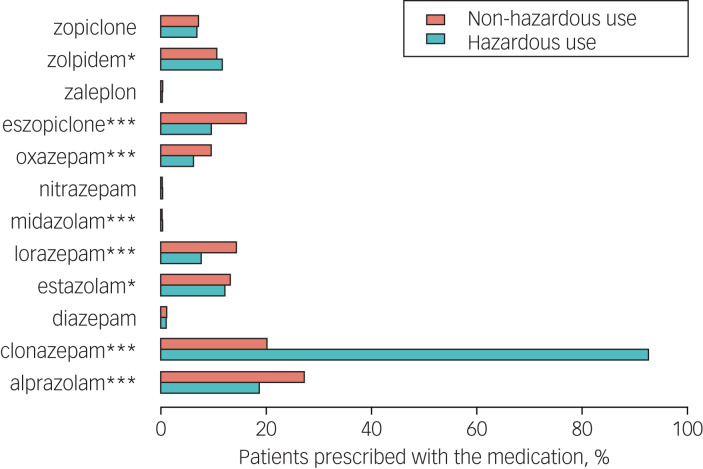
Percentage of patients who were medicated by a specific hypnotic in the hazardous and non-hazardous use groups. *P < 0.05, ***P < 0.001.

As shown in Table 2, the largest proportion of patients were prescribed BZDs only (67.9%), followed by Z-drugs only (21.8%); a small proportion were treated with both BZDs and Z-drugs (10.3%). We found that BZD consumption was much higher than Z-drug consumption in general. The DDD for all types of hypnotic was relatively low in hospitals D and E (the central region), whereas the DDD for BZDs only was highest in hospital C (the west region) and the DDD for Z-drugs was relatively high in hospitals A and B (the east region). Specifically, clonazepam and alprazolam were predominantly used, whereas zaleplon, nitrazepam and midazolam were much less commonly prescribed (Fig. 4).

**Table 2:** Overall use of benzodiazepine receptor agonists in five hospitals in China

Variables	Total sample (*n* = 164 450)	Hospital A (*n* = 49 121)	Hospital B (*n* = 17 851)	Hospital C (*n* = 72 161)	Hospital D (*n* = 13 756)	Hospital E (*n* = 1 1561)
BZDs only, *n* (%)	111 613 (67.9%)	30 080 (61.2%)	10 864 (22.1%)	51 935 (72.0%)	9058 (65.8%)	9676 (83.7%)
Z-drugs only, *n* (%)	35 895 (21.8%)	12 127 (24.7%)	4293 (8.7%)	14 802 (20.5%)	3426 (24.9%)	1247 (10.8%)
BZDs + Z-drugs, *n* (%)	16 942 (10.3%)	6914 (14.1%)	2694 (5.5%)	5424 (7.5%)	1272 (9.2%)	638 (5.5%)
DDD (BZDs), mean	11.62	10.57	12.09	14.54	5.75	4.03
DDD (Z-drugs), mean	5.88	8.14	9.83	5.05	0.78	1.56
DDD (BZDs + Z-drugs), mean	17.51	18.72	21.92	19.59	7.34	5.59

BZDs, benzodiazepines; DDD, defined daily dose.

### Comparison between hazardous users and non-hazardous users

We further compared characteristics and BZRA prescription patterns of hazardous users with those of non-hazardous users (Table 3). Compared with non-hazardous users, hazardous users were more likely to be male (40.7% *v*. 36.2%, *P* < 0.001) and older than 65 years (28.1% *v*. 20.9%, *P* < 0.001).

**Table 3:** Characteristics and prescription patterns: comparison between hazardous users and non-hazardous users

Variables	Hazardous users (*n* = 4988)	Non-hazardous users (*n* = 159 462)	*P*
Gender, *n* (%)			0.000
Male	2030 (40.7%)	57 683 (36.2%)	
Female	2949 (59.1%)	101 696 (63.8%)	
Age, years: *n* (%)			0.000
<18	19 (0.4%)	3632 (2.3%)	
18–64	3516 (70.5%)	121 877 (76.4%)	
>=65	1404 (28.1%)	33 310 (20.9%)	
Over-indication use, *N* (%)	3712 (74.4%)	88 223 (55.3%)	0.000
Maximum average daily DME usage, median (IQR)	59.56 (47.21–87.12)	4.27 (1.67–12.76)	0.000
Total DME dosage, median (IQR)	13 920 (10211.25–20000)	453 (157.5–1830)	0.000
Maximum continuous days of use, median (IQR)	142 (111–197.25)	33 (23–50)	0.000
Total days of use, median (IQR)	282 (214–322)	40 (20–100)	0.000
Number of out-patient visits, median (IQR)	12 (9–14)	3 (1–6)	0.000
Number of different doctors visited, median (IQR)	7 (5–10)	2 (1–4)	0.000
ICD-10 diagnosis codes, *n* (%)			
F01–F09	131 (2.6%)	3917 (2.5%)	0.446
F10–F19	41 (0.8%)	751 (0.5%)	0.000
F20–F29	918 (18.4%)	14252 (8.9%)	0.000
F30–F39	2178 (43.7%)	51661 (32.4%)	0.000
F40–F49	1375 (27.6%)	53759 (33.7%)	0.000
F50–F59	216 (4.3%)	4825 (3.0%)	0.000
F60–F69	4 (0.1%)	208 (0.1%)	0.330
F70–F79	20 (0.4%)	274 (0.2%)	0.000
F80–F89	0 (0.0%)	36 (0.0%)	0.565
F90–F98	7 (0.1%)	538 (0.3%)	0.017
F99–F99	322 (6.5%)	8532 (5.4%)	0.001
G40–G41	161 (3.2%)	1474 (0.9%)	0.000
G47	1055 (21.2%)	45265 (28.4%)	0.000

F01–F09: Organic, including symptomatic, mental disorders; F10–F19: Mental and behavioral disorders due to psychoactive substance use; F20–F29: Schizophrenia, schizotypal and delusional disorders; F30–F39: Mood [affective) disorders; F40–F49: Neurotic, stress-related and somatoform disorders; F50–F59: Behavioral syndromes associated with physiological disturbances and physical factors; F60–F69: Disorders of adult personality and behavior; F70–F79: Mental retardation; F80–F89: Disorders of psychological development; F90–F98: Behavioral and emotional disorders with onset usually occurring in childhood and adolescence; F99–F99: Unspecified mental disorder; G40–G41: Epilepsy and recurrent seizures; G47: Sleep disorders; DME, diazepam milligram equivalent.

Among hazardous users, 74.4% had over-indication use. Their maximum averaged daily DME usage, total DME dosage, maximum continuous days of use and total days of use were all greater than those observed in non-hazardous users. Further, hazardous users had more out-patient visits and visited a higher number of different doctors than non-hazardous users. Significant differences were found between hazardous and non-hazardous users for all diagnoses except for F01–F09 (Organic, including symptomatic, mental disorders), F60–F69 (Disorders of adult personality and behaviour) and F80–F89 (Disorders of psychological development). The three most common diagnoses were codes F30–F39 (43.7%), F40–F49 (27.6%) and G47 (21.2%) in hazardous users, and F30–F39 (32.4%), F40–F49 (33.7%) and G47 (28.2%) in non-hazardous users.

We also compared the specific medications prescribed to hazardous and non-hazardous users. Hazardous users were prescribed more clonazepam, midazolam and zolpidem than non-hazardous users, whereas non-hazardous users received more prescriptions for eszopiclone, oxazepam, lorazepam, estazolam and alprazolam (Fig. 4).

### Factors associated with hazardous use

In this multilevel multivariate regression model, 56 983 observations were deleted owing to missingness. Although older age and male gender were significantly associated with high risk of hazardous BZRA use, region variables had no significant effect (Table 4). Moreover, over-indication use, more frequent out-patient visits and greater number of different doctors visited also predicted the risk of hazardous BZRA use.

**Table 4:** Multilevel regression predicting hazardous use of benzodiazepine receptor agonists (*n* = 163 720).

Variables	Coefficient β	95% CI	*P*
Age, years			
<18	Ref.		
18–64	0.018	0.013 to 0.023	<0.001
≥65	0.015	0.010 to 0.021	<0.001
Gender			
Female	Ref.		
Male	0.005	0.003 to 0.007	<0.001
Region of China			
East	Ref.		
Central	0.007	−0.005 to 0.019	0.227
West	0.008	–0.007 to 0.022	0.278
Over-indication use (yes)	0.013	0.012 to 0.015	<0.001
Number of out-patient visits	0.006	0.006 to 0.006	<0.001
Number of different doctors visited	0.007	0.007 to 0.008	<0.001

Ref., reference.

### The most common drugs for over-indication use and their corresponding diagnoses

Among patients with over-indication use, the three most commonly prescribed drugs were clonazepam (32.9%), alprazolam (18.3%) and eszopiclone (13.4%). The most common diagnoses that did not meet indications for clonazepam were codes F30–F39 (66.6%), F40–F49 (27.7%) and G47 (19.0%). The most common diagnoses that did not meet alprazolam indications were codes F30–F39 (64.9%), F20–F29 (19.1%) and F99–F99 (7.7%). The most common diagnoses for eszopiclone over-indication were codes F30–F39 (51.4%), F40–F49 (26.1%) and F20–F29 (10.2%).

### Patient follow-up

In one of the hospitals in the east region 98.5% of patients with hazardous use could not be effectively contacted owing to missing or wrong contact information recorded in the out-patient prescription system (72.5%) or unwillingness to be followed up (26.0%). Only three respondents (1.5%) completed the interview, one of whom met the DSM-5 criteria for what might be called a sedative or hypnotic dependence syndrome.

## Discussion

In this study, we examined the real-world prescription patterns for hazardous BZRA use and its related factors in psychiatric settings using electronic prescription dispensing data from five hospitals in the east, central and west China. The study implied that: (a) the majority of patients were medicated with relatively safe doses and duration, but the small proportion of extreme individuals should be identified; (b) medication prescribing patterns varied among different hospitals and regions; (c) factors associated with a greater likelihood of hazardous use were being male, older age, a higher number of out-patient visits and a higher number of different doctors visited. There is a lack of direct comparability with previous data owing to variations in database sources, study populations, years investigated and methodological approaches. To the best of our knowledge, this was the first large-scale population-based study to investigate BZRA prescriptions among Chinese psychiatric out-patients.

BZRA prescription was found to be highest among women aged 18–64 (76.2%) in psychiatric out-patient settings, which was consistent with findings of a study in urban China between 2013 and 2017.^21^ The top three diagnoses for which hypnotics were prescribed were mood disorder, neurotic, stress-related and somatoform disorders, and sleep disorders, which was an expected result as BZRAs are primarily prescribed for insomnia and anxiety.^3^ People with mood disorders usually show sleep disturbances or anxiety, so BZRAs are often recommended as adjunctive drugs for treatment in clinical practice.^22^ The higher BZRA prescription rate among women is probably related to a higher prevalence of anxiety disorders, affective disorders and sleep disorders among females compared with males.^23,24^ However, there is a lack of empirical research concerning the potential benefits or harms of hypnotics when used with antidepressants,^25^ which deserves special attention.

In our observation period in 2018, patients’ median maximum average daily DME usage was 4.4 and the median maximum continuous days of use was 33 days, indicating that the majority of patients were treated with relatively low doses and short-term use, as defined in this study. A nationwide and longitudinal study in Japan also found that long-term use of hypnotic medications was uncommon,^26^ whereas a study in England showed that long-term (>12 months) prescribing was common.^9^ Only 4988 individuals (3.0%) who showed a hazardous pattern of BZRA use were identified. However, there was still a small proportion of patients with extremely overdose or long-term use in hospital E, as some clinicians may have prescribed in this way in response to patient's requests to reduce the number of hospital visits, which warrants attention.

With regard to specific drugs, we found that the percentage of BZD prescriptions is much higher than Z-drug prescriptions, with clonazepam and alprazolam being the most commonly prescribed, indicating that psychiatrists in China seem to prefer BZDs to Z-drugs for hypnotic prescriptions. The reason for the popularity of clonazepam in psychiatric settings may be due to its long half-life.^27^ It is worth noting that clonazepam is a high-potency BZD which may easily cause addiction and it was found to be the BZD most commonly associated with dose increase in a recent Canadian study.^28^

BZRA prescribing patterns varied among the different hospitals and regions in this study. Overdose and long-term use in the central region were relatively less common than in the other regions. Moreover, patients in hospitals A and B, which are located in the east of China, had a higher number of out-patient visits and saw a greater number of different doctors compared with those in the hospitals in central and west China. This may be explained by the fact that local governmental regulations for BZRA prescription in the central region are much stricter. The largest proportion of hazardous users was found in the east region. People living in east China are considered to have higher economic status and suffer much higher social pressure compared with those in central and west China.^29^ This may increase the prevalence of insomnia (including that caused by anxiety and depression), thereby increasing the demand for medicine.^30^ Overall, a varieties of factors might account for this trend of differing prescribing patterns between hospitals and regions, such as the local healthcare system, socioeconomic status, prescription habits of psychiatrists and other factors that vary from region to region.^29^ Future work is needed to optimise and standardise pharmaceutical administration among different sites.

In line with a previous study,^18^ factors associated with hazardous use were male gender and older age. Male patients often behave more impulsively and aggressively than women patients,^20^ so male patients were more likely to use BZRAs hazardously. Olfson et al^31^ have pointed out the lack of promising alternative treatments for sleep disorders and noted that older patients were more likely to lack the motivation to discontinue or reduce the use of BZDs. Off-label usage was associated with higher risk of hazardous use and 55.9% of patients in our study had over-indication prescriptions. We found that clonazepam, alprazolam and eszopiclone were the three drugs most commonly prescribed over-indication and the recorded diagnoses were mainly mood disorders and schizophrenia. There are a number of potential reasons that may explain this trend. First, some psychiatrists lacked standardised operations for clinical prescriptions, so patient's diagnoses may be wrongly or not fully recorded in the prescription system. Second, there may be a lag between drug indications approved by the Chinese FDA and the newest clinical guidelines: for example, clonazepam has an anxiolytic effect, but the instruction manual only indicates its use in treatment for epilepsy. Third, variance in drug indications between China and other countries may also be a contributory factor. Our results highlight the great need that guidelines should be more in accordance with practical needs. An improved out-patient prescription system and enhanced professional training for psychiatrists are also required.

Our data also suggested that hazardous use was associated with more frequent out-patient visits and visiting a greater number of different doctors. Doctor shopping, defined as repeated visits to different doctors, has been associated with misuse of prescription drugs.^32^ A survey conducted in the USA found that 4.2% of hypnotics users had received a prescription from different doctors within a 30 day period.^19^ However, in one hospital in our study most of the patients with hazardous BZRA use (98.5%) refused to be followed up or were not contactable because of invalid telephone numbers. If the trajectories of hypnotics cannot be traced, it may increase the risks of drug misuse, dependence and complications. Future work should be done to improve drug monitoring and management systems for better follow-up.

### Limitations

The study has several limitations. First, the findings are not necessarily representative of the situation in rural areas or small-scale hospitals (such as local community hospitals or clinics) in China as the hospitals chosen were tertiary hospitals in urban areas. However, BZRAs might be less prescribed in rural areas or primary hospitals owing to limited treatment levels or constrained resources. One study found that patients from an urban area in China were significantly more frequently prescribed antipsychotic polypharmacy.^33^ Therefore, we could consider that the sampled hospitals with the largest number of out-patient visits within their own regions could well represent psychiatric out-patient situations in China to some extent. Second, we conducted the analysis using prescription records derived from electronic databases, which may not indicate the amount of BZRAs that patients take in the real world. Patients may have received BZRAs from friends, family members or outside the sampled hospitals. Consequently, the number of hazardous users would be underestimated. Third, as the definition of hazardous and over-indication use was relatively conservative in this study, the true proportion of these prescription patterns might be higher.

### Implications and future research

This study was designed to use prescription databases to identify hazardous BZRA use, which has important implications for detecting potential patients with hypnotics dependence and providing early intervention. Future research should continue to focus on identifying hazardous users using prescription data, for example by creating prediction models using machine learning algorithms, or identifying features related to hazardous use for early diagnosis in clinical practice.

## Data Availability

The prescription data in this study are not available for sharing.

## Supplementary material

For supplementary material accompanying this paper visit http://doi.org/10.1192/bjo.2022.589. click here to view supplementary material
