# Hazardous use of benzodiazepine receptor agonists in psychiatric clinics in China: electronic prescription database study – CORRIGENDUM

**DOI:** 10.1192/bjo.2022.609

**Published:** 2022-11-10

**Authors:** Xiaomin Xu, Xuyi Wang, Na Zhong, Jiajun Xu, Chuanwei Li, Gang Wang, Wenzhe Wang, Yujian Ye, Yong Chen, Tieqiao Liu, Min Zhao, Haifeng Jiang

The authors regret the inclusion of an error in the above article.

The original order of the author list and number of joint first authors was incorrect, published as follows:

Xiaomin Xu*, Jiajun Xu*, Chuanwei Li*, Gang Wang*, Wenzhe Wang, Yujian Ye, Yong Chen, Tieqiao Liu, Min Zhao, Xuyi Wang, Na Zhong and Haifeng Jiang

*Joint first authors

The updated author list is as follows:

Xiaomin Xu*, Xuyi Wang*, Na Zhong*, Jiajun Xu*, Chuanwei Li*, Gang Wang*, Wenzhe Wang, Yujian Ye, Yong Chen, Tieqiao Liu, Min Zhao and Haifeng Jiang

*Joint first authors

The Author contribution statement has also been amended as follows:

X.X., X.W., N.Z., J.X., G.W. and C.L. are co-first authors and contributed equally to writing the manuscript. H.J., N.Z. and X.W. are the principal investigators who conceived the original idea, implemented and analysed the data for the study and X.W., J.X., G.W., C.L. and Y.Y. are co-principal investigators who made contributions to the trial design and implementation. M.Z. and T.L. supervised the study design and data analysis. X.X. and W.W. did the coding for data cleaning and data analysis. Y.C. contributed to the revision of the manuscript and suggestions for data analysis. All authors contributed to the interpretation of the data, and critically reviewed the content and approved the final version for publication.

The article has been corrected.

