# Supplementary material for: Hazardous use of benzodiazepine receptor agonists in psychiatric clinics in China: electronic prescription database study
Source: BJPsych Open. 2022 Oct 18;8(6):e188. doi: 10.1192/bjo.2022.589 (PMC9634586; doi:10.1192/bjo.2022.589)
Supplement: Supplementary file 1 [file S2056472422005890sup001.docx]

**Supplementary material**

**Table S1. Indications of benzodiazepine receptor agonists approved by the Chinese Food and Drug Administration.**

| Drug | Anxiolytic effect | Sedative effect | Hypnotic effect | Treatment for alcohol withdrawal | Treatment for panic disorder | Treatment for epilepsy | Anesthetic effect |
| --- | --- | --- | --- | --- | --- | --- | --- |
| Alprazolam | √ | √ | √ | × | √ | × | × |
| Estazolam | × | √ | √ | × | × | √ | × |
| Lorazepam | √ | × | √ | × | × | × | × |
| Oxazepam | √ | √ | √ | √ | × | × | × |
| Midazolam | × | √ | × | × | × | √ | √ |
| Diazepam | √ | √ | √ | √ | × | × | √ |
| Clonazepam | × | × | × | × | × | √ | × |
| Nitrazepam | × | × | √ | × | × | √ | × |
| Zolpidem | × | × | √ | × | × | × | × |
| Zopiclone | × | × | √ | × | × | × | × |
| Eszopiclone | × | × | √ | × | × | × | × |
| Zaleplon | × | × | √ | × | × | × | × |
